# Supplementary figures and images for: Structure of the Helicobacter pylori Cag type IV secretion system
Source: eLife. 2019 Jun 18;8:e47644. doi: 10.7554/eLife.47644 (PMC6620104; doi:10.7554/eLife.47644)

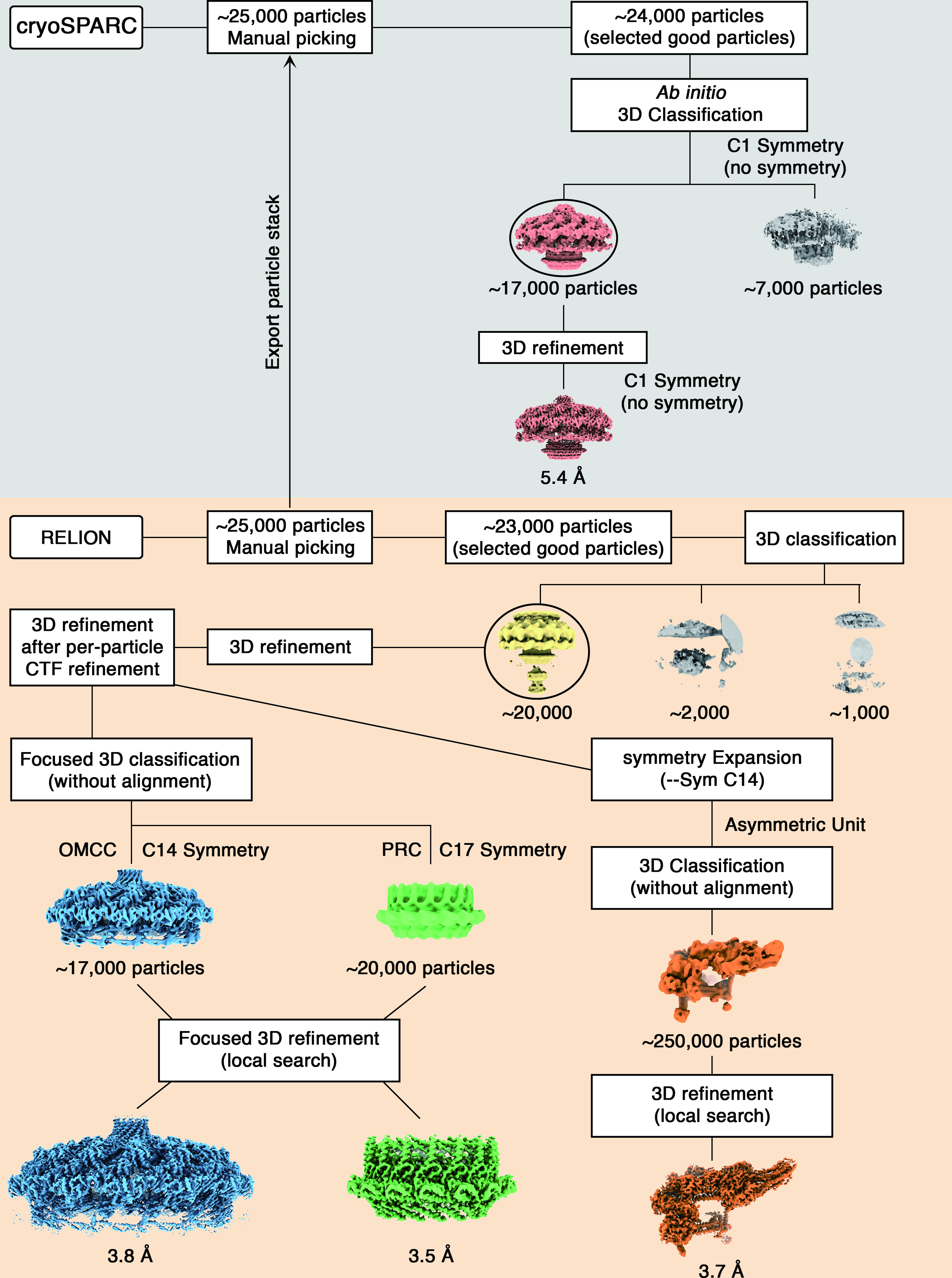

Supplement: Supplementary file 1. — ~25,000 Cag T4SS particles were manually picked in RELION and analyzed by two different image processing software packages (CryoSPARC and RELION). The processing steps done in CryoSPARC are on a gray background and the processing steps done using RELION are on a tan background. The particles were exported into cryoSPARC for 2D alignment and ab initio 3D classification. The best class was chosen for further refinement, without symmetry (C1). The 5.4 Å 3D model with no symmetry applied was filtered to 60 Å resolution and used as an initial model for 3D structure determination in RELION using 3D refinement without applied symmetry. Focused 3D classification (without alignment) was used to determine higher resolution maps of the OMCC (with 14-fold symmetry) and the PRC (with 17-fold symmetry). The maps of the OMCC and PRC were further refined using focused 3D refinement (with local refinement), resulting in 3D maps of the OMCC (14-fold symmetry) and the PRC (17-fold symmetry) at 3.8 Å and 3.5 Å, respectively. To improve the resolution of the OMCC, symmetry expansion was applied, resulting in the 3D reconstruction at 3.7 Å resolution. [file elife-47644-supp1.jpg]

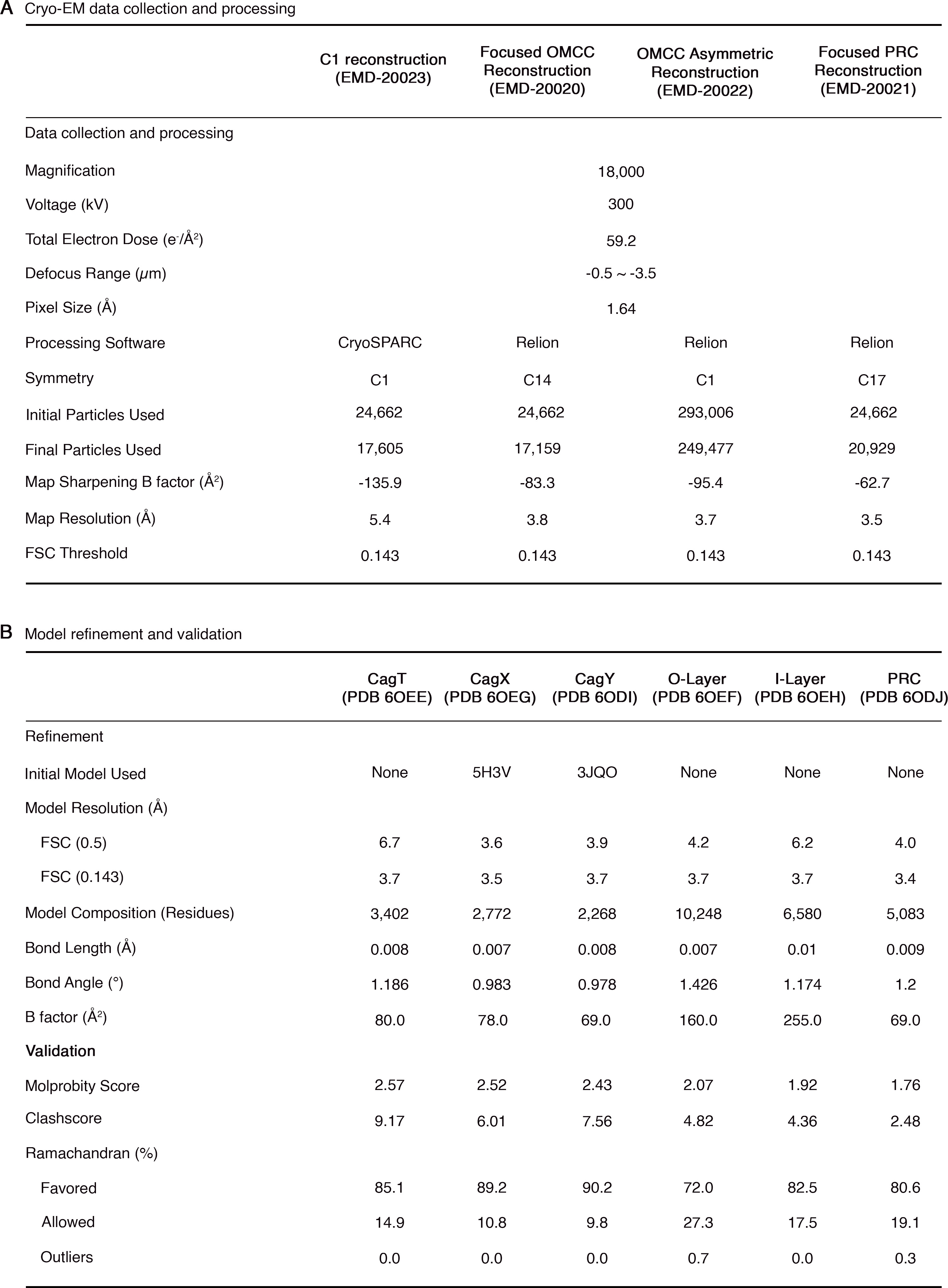

Supplement: Supplementary file 2. — (A) Information about the cryo-EM data collection and data sets. (B) Information about model and model validation. [file elife-47644-supp2.jpg]

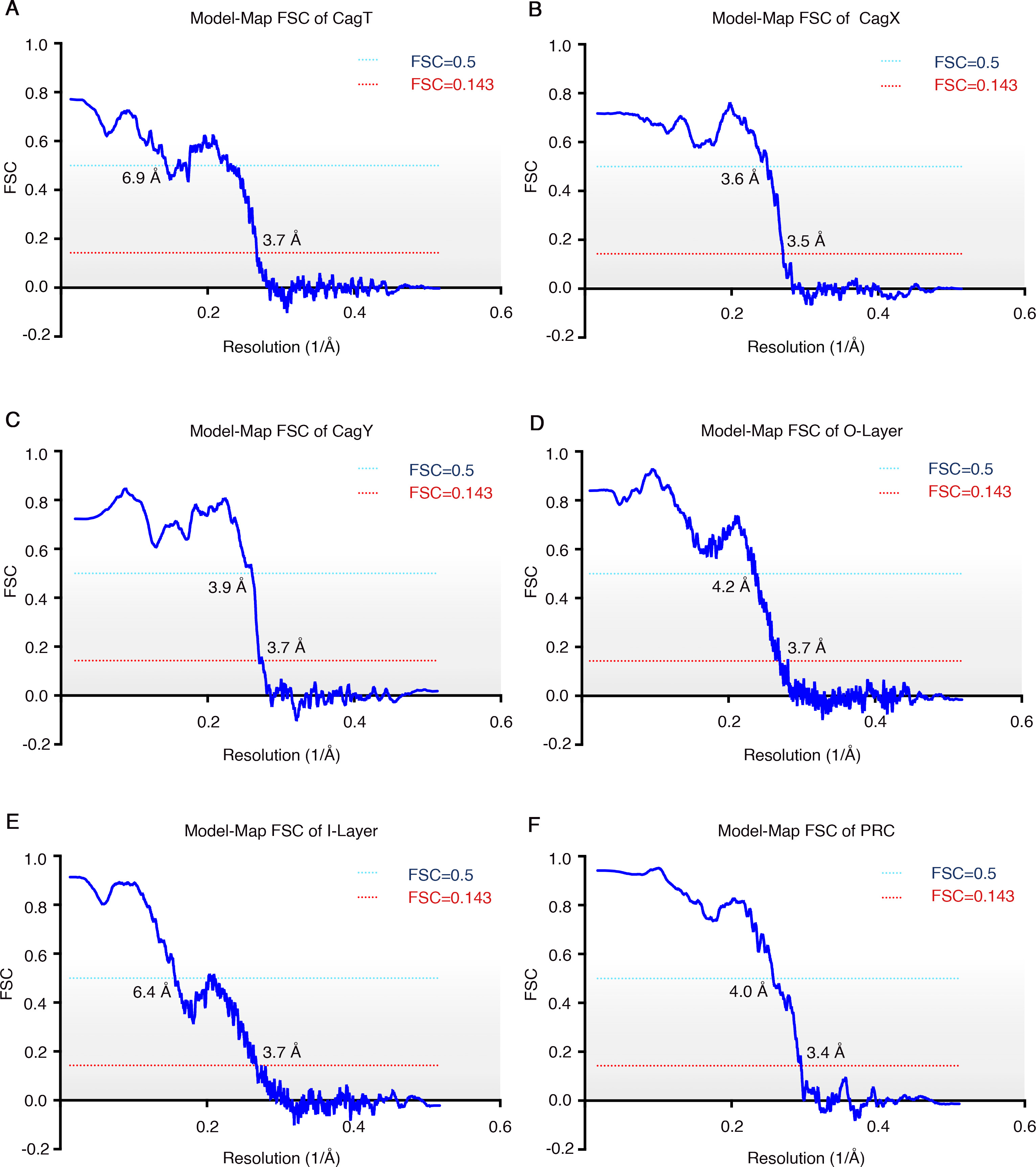

Supplement: Supplementary file 3. — A-F. FSC of the half map against the refined model of CagT (A), CagX (B), CagY (C), O-layer (D), I-layer (E), and PRC (F). [file elife-47644-supp3.jpg]
