# Supplementary material for: Structure of the Helicobacter pylori Cag type IV secretion system
Source: eLife. 2019 Jun 18;8:e47644. doi: 10.7554/eLife.47644 (PMC6620104; doi:10.7554/eLife.47644)
Supplement: Transparent reporting form [file elife-47644-transrepform.docx]

***eLife’s* transparent reporting form**

We encourage authors to provide detailed information *within their submission* to facilitate the interpretation and replication of experiments. Authors can upload supporting documentation to indicate the use of appropriate reporting guidelines for health-related research (see [EQUATOR Network](http://www.equator-network.org/%20)), life science research (see the [BioSharing Information Resource](https://biosharing.org/)), or the [ARRIVE guidelines](http://www.plosbiology.org/article/info:doi/10.1371/journal.pbio.1000412) for reporting work involving animal research. Where applicable, authors should refer to any relevant reporting standards documents in this form.

If you have any questions, please consult our Journal Policies and/or contact us: [editorial@elifesciences.org](mailto:editorial@elifesciences.org).

**Sample-size estimation**

- You should state whether an appropriate sample size was computed when the study was being designed
- You should state the statistical method of sample size computation and any required assumptions
- If no explicit power analysis was used, you should describe how you decided what sample (replicate) size (number) to use

Please outline where this information can be found within the submission (e.g., sections or figure legends), or explain why this information doesn’t apply to your submission:

Our sample size was determined by the amount of high quality images we were able to collect on the Titan Krios. This data set was sufficient to determine ~3.5 Å structure of the H. pylori Cag T4SS. Information about sample size and how the data was collected is found in the Methods Section, Figure 1-figure supplement 1, Figure 1-figure supplement 2, and Supplementary File 1.

**Replicates**

- You should report how often each experiment was performed
- You should include a definition of biological versus technical replication
- The data obtained should be provided and sufficient information should be provided to indicate the number of independent biological and/or technical replicates
- If you encountered any outliers, you should describe how these were handled
- Criteria for exclusion/inclusion of data should be clearly stated
- High-throughput sequence data should be uploaded before submission, with a private link for reviewers provided (these are available from both GEO and ArrayExpress)

Please outline where this information can be found within the submission (e.g., sections or figure legends), or explain why this information doesn’t apply to your submission:

The structure was determined using established cryo-EM image processing and validation methods. We determined the resolution of our structure using the FSC Gold Standard. The resolution of each individual model was estimated by Fourier Shell correlation against the map used to construct it within the Phenix Cryo-EM Validation tool (Figure 1-figure supplement 1 and 2 and Figure 4-figure supplement 2). Molprobity scores, Clashscores and Ramachandran plots were used to validate the models that were constructed (Supplementary File 2). Supplementary File 3 shows the FSCs of the half maps against the refined model agree with each other, suggesting that the models are not over-refined.

**Statistical reporting**

- Statistical analysis methods should be described and justified
- Raw data should be presented in figures whenever informative to do so (typically when N per group is less than 10)
- For each experiment, you should identify the statistical tests used, exact values of N, definitions of center, methods of multiple test correction, and dispersion and precision measures (e.g., mean, median, SD, SEM, confidence intervals; and, for the major substantive results, a measure of effect size (e.g., Pearson's r, Cohen's d)
- Report exact p-values wherever possible alongside the summary statistics and 95% confidence intervals. These should be reported for all key questions and not only when the p-value is less than 0.05.

Please outline where this information can be found within the submission (e.g., sections or figure legends), or explain why this information doesn’t apply to your submission:

This manuscript reports the structure of the H. pylori Cag T4SS determined using single particle cryo-EM. No statistical reporting was required for these studies. We did use the Gold-Standard FSC criteria to determine the resolution of our cryo-EM model. We also validated the models built from the cryo-EM map using Phenix, Molprobity scores, Clashscores and Ramachandran plots. This information can be found in Figure 1-figure supplement 1 and 2 and Figure 4-figure supplement 2, as well as, Supplemental Files 2 and 3.

(For large datasets, or papers with a very large number of statistical tests, you may upload a single table file with tests, Ns, etc., with reference to sections in the manuscript.)

**Group allocation**

- Indicate how samples were allocated into experimental groups (in the case of clinical studies, please specify allocation to treatment method); if randomization was used, please also state if restricted randomization was applied
- Indicate if masking was used during group allocation, data collection and/or data analysis

Please outline where this information can be found within the submission (e.g., sections or figure legends), or explain why this information doesn’t apply to your submission:

Particles in our data set were sorted by 2D and 3D averaging during data processing. We used focused refinement to improve the resolutions of sub-complexes in the T4SS. The summary of this process and how these decisions were made are described in the methods section and shown in Supplemental Files 1 and 2.

**Additional data files (“source data”)**

- We encourage you to upload relevant additional data files, such as numerical data that are represented as a graph in a figure, or as a summary table
- Where provided, these should be in the most useful format, and they can be uploaded as “Source data” files linked to a main figure or table
- Include model definition files including the full list of parameters used
- Include code used for data analysis (e.g., R, MatLab)
- Avoid stating that data files are “available upon request”

Please indicate the figures or tables for which source data files have been provided:

We have submitted all our cryo-EM maps and PDB coordinates for the review process. The maps and models have also been deposited in the EMDB and PDB.
